# Hydrostatin-TL1, an Anti-Inflammatory Active Peptide from the Venom Gland of *Hydrophis cyanocinctus* in the South China Sea

**DOI:** 10.3390/ijms17111940

**Published:** 2016-11-22

**Authors:** Ningyuan Wang, Yan Huang, An Li, Hailong Jiang, Jie Wang, Jianzhong Li, Lei Qiu, Ka Li, Yiming Lu

**Affiliations:** 1 School of Pharmacy, Fujian University of Traditional Chinese Medicine, Fuzhou 350108, China; wangningyuan1230@163.com; 2 Department of Biochemical Pharmacy, School of Pharmacy, Second Military Medical University, Shanghai 200433, China; huangyantiger2005@163.com (Y.H.); ezioleon@foxmail.com (A.L.); jhlong1986@163.com (H.J.); wj890801@126.com (J.W.); lijianzhong1234@hotmail.com (J.L.); qlcong021@163.com (L.Q.); 3 Biomedical Research Center, Zhongshan Hospital, Fudan University, Shanghai 200032, China

**Keywords:** H-TL1, *Hydrophis cyanocinctus*, anti-inflammatory, inflammatory bowel diseases, TNF-α

## Abstract

Tumor necrosis factor (TNF)-α is a pleiotropic cytokine with intense pro-inflammatory and immunomodulatory properties, and anti-TNF-α biologics are effective therapies for various inflammatory diseases such as inflammatory bowel disease (IBD) and sepsis. Snake venom, as a traditional Chinese medicine, has been used in the treatment of inflammatory diseases in China for centuries. In this research, we constructed a venom gland T7 phage display library of the sea snake *Hydrophis cyanocinctus* to screen bioactive compounds that antagonize TNF-α and identified a novel nine-amino-acid peptide, termed hydrostatin-TL1 (H-TL1). In enzyme-linked immunosorbent assay (ELISA) and surface plasmon resonance (SPR) analyses, H-TL1 inhibited the interaction between TNF-α and TNF receptor 1 (TNFR1). Further, H-TL1 attenuated the cytotoxicity of TNF-α in L929 cells as determined by the 3-(4,5-dimethylthiazol-2-yl)-2,5-diphenyl tetrazolium bromide (MTT) assay. H-TL1 also decreased the mRNA expression of TNF-α/TNFR1 downstream targets and suppressed the phosphorylation of well-characterized proteins of downstream signal transduction pathways in HEK-293 cells. In vivo data demonstrated that H-TL1 protects animals against dextran sodium sulfate (DSS)-induced acute colitis and lipopolysaccharide (LPS)-induced acute shock. Given its significant anti-inflammatory activity in vitro and in vivo, H-TL1 is a potential peptide for the development of new agents to treat TNF-α-associated inflammatory diseases.

## 1. Introduction

Tumor necrosis factor (TNF-α, or TNF) is a major mediator of apoptosis as well as inflammation and immunity, and it has been implicated in the pathogenesis of a wide spectrum of human diseases such as diabetes, cancer, osteoporosis, multiple sclerosis, rheumatoid arthritis, acute shock, and inflammatory bowel disease (IBD) [1]. As a pleiotropic cytokine, an increase in TNF-α expression is a benchmark of the inflammatory response. TNF-α is synthesized as a transmembrane protein and can be processed by TNF-α converting enzyme into its soluble form, which exerts its biological functions by acting on two TNF-α receptors (TNFR), TNFR1 and TNFR2 [2]. TNFR1, which contains a death domain in its cytoplasmic tail, appears to play important pro-inflammatory, cytotoxic, and apoptotic roles [3]. In contrast to TNFR1, signaling through TNFR2 has been shown to be anti-inflammatory and might be involved in specific anti-infective defenses [4,5,6]. Therefore, exploring the inhibitory mechanisms underlying interactions between TNF-α and TNFR1 is an important strategy for developing treatments of inflammation-related diseases.

In recent years, antibodies (such as infliximab, adalimumab, and golimumab) against TNF-α have been approved for patient use [7]; however, a lack of cost-effectiveness and inconvenient route of administration limit their long-term use in patients without sufficient financial support. To reduce the cost to patients, recent research studies have focused on discovering small molecules that directly inhibit the binding of TNF-α to TNFR1 [8,9]. The current basic and applied research into TNF-α inhibitors has been rather unsatisfactory because they require a high effective concentration, are relatively insoluble, and have serious side effects [10]. Moreover, it has been suggested that small molecules are insufficient for disrupting the TNF-α/TNFR1 complex because of its particularly large interaction surface [11]. Compared to small molecular inhibitors, bioactive peptides have sufficient volume and disulfide bonds, which are important for their structure, function, and stability, as well as for enabling adequate embedment onto the surface of the protein interaction complex. 

Phage display has emerged as a powerful high-throughput screening tool that can rapidly link natural product ligands to a variety of specific cellular targets, including enzymes and membrane receptors [12]. In the T7 phage display system, a gene encoding a peptide of interest is inserted into the genome of bacteriophage T7 and then transduced into *Escherichia coli* (*E. coli*) cells, resulting in the peptide sequence fused to the C-terminus of the 10B capsid protein and expressed on the surface of the phage particles [13]. These displaying phages can then be screened against target proteins that are immobilized onto the surface of microtiter plate wells to detect the ligand-receptor interaction. In this way, large libraries of peptides are capable of being presented on the phage surface and panned in a process comprising repeated cycles of binding, washing, elution, and amplification. Thereafter, by sequencing the genomes of the increasingly enriched phages, the displayed peptide sequences can be obtained and reproduced as recombinant or synthetic peptides; finally, a unique binder with high affinity and specificity for desired targets can be identified [14]. Phage display peptide libraries usually contain up to 10^10^ diverse variants [12] that enable peptides to appear with an extensive variety of sizes and structures on the phage surface. Natural peptides directly isolated using traditional separation methods, including high-performance liquid chromatography (HPLC), are generally present at relatively low concentrations in complex mixtures of biological components. In contrast, phage displays represent a more economical and efficient alternative for the selection of specific peptide ligands interacting with inflammatory mediators [15,16]. 

Snake-derived products have been used as traditional Chinese medicines for centuries in China; however, their mechanisms of action and active constituents are still unknown. Snake venom, which is a mixture of proteins and peptides, has various biological properties including antitumor, anti-inflammatory, anti-stroke, and analgesic activities [17]. Cathelicidin-BF (C-BF), a new cathelicidin peptide purified from *Bungarus fasciatus* snake venom, has shown both antimicrobial and anti-inflammatory properties. Moreover, C-BF decreases the expression and secretion of TNF-α [18,19]. Venom from sea snakes is more streamlined and stable than that from land snakes [20], and *Hydrophis cyanocinctus*, a primary sea snake species in China, has been used to treat rheumatism for centuries. Wei et al. reported a novel cathelicidin (Hc-CATH) from the *H. cyanocinctus* sea snake that exhibits potent anti-inflammatory activity by inhibiting pro-inflammatory cytokines such as TNF-α, IL-1β, and IL-6 [21]. Furthermore, hydrostatin-SN1, which was identified by screening a *H. cyanocinctus* venom gland T7 phage display library, exhibited significant anti-inflammatory activity in a DSS-induced acute colitis mouse model [22]. These studies further support the notion that screening sea snake venom for compounds with anti-inflammatory properties is a potentially useful strategy for developing new drug candidates.

In summary, the aim of this study was to identify and screen new peptides from an *H. cyanocinctus* venom gland T7 phage display library with bioactivity against TNF-α. We constructed and evaluated biological functions of the peptides in vitro and in vivo. Subsequently, we identified hydrostatin-TL1 (H-TL1) as an anti-inflammatory peptide with significant TNF-α binding activity and further evaluated its in vitro and in vivo effects using relevant models. Construction and biopanning of a *H. cyanocinctus* venom gland T7 phage display library provide a novel approach for screening candidate peptides that are related to protein interactions.

## 2. Results and Discussion

### 2.1. Biopanning and Sequencing

**Figure 1:**
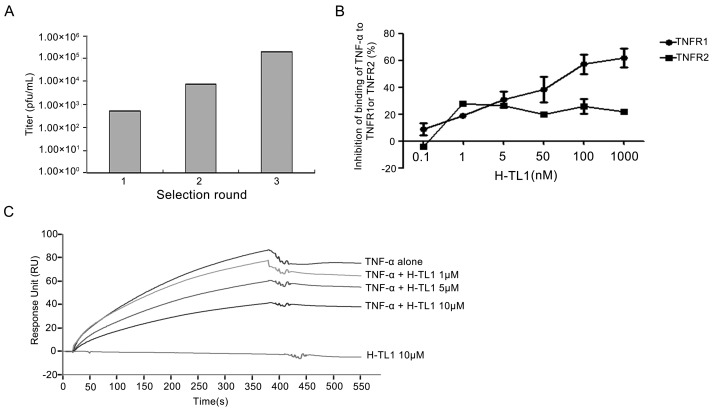
Biopanning and identification: (**A**) The titer increase of specifically bound phages after each selection; (**B**) ELISA assay showing that H-TL1 obviously inhibits the binding of TNF-α with TNFR1, but had little impact on TNF-α/TNFR2 interactions. Values represent means ± SEM (*n* = 3); and (**C**) SPR analysis indicating that H-TL1 inhibits the association of TNF-α with sensor chip-immobilized TNFR1.

The *H. cyanocinctus* venom gland T7 phage display library was constructed with an original titer of 1.56 × 10^6^ pfu/mL. The biopanning process comprised three rounds of selection on TNF-α-coated plates. After each round of selection, the eluted phages were amplified in *E. coli* BLT5403, and the titers of the eluted buffer and amplified phages were determined. The eluted phages were enriched from 10^2.7^ to 10^5.3^ (Figure 1A) after three rounds of selection. We selected one potential binding peptide after sequencing and termed it H-TL1; its nucleotide and amino acid sequences are 5′-GCAACTTCAAAGCCAAGCCTCAAGTGT-3′ and Ala-Thr-Ser-Lys-Pro-Ser-Leu-Lys-Cys-COOH (ATSKPSLKC-COOH), respectively.

### 2.2. Competitive Inhibition Assays

We investigated whether H-TL1 has an inhibitory effect on the binding of TNF-α to TNFRs by using ELISA. In this assay, a fixed amount of TNF-α was mixed with different concentrations of H-TL1 and applied to the TNFR-coated ELISA plates. As shown in Figure 1B, H-TL1 inhibited the binding of TNF-α with TNFR1 in a concentration-dependent manner within the range of 0.1–1000 nM; high concentrations (1 μM) significantly inhibited the binding by nearly 60%. Although H-TL1 had some effect on the binding of TNF-α with TNFR2 (approximately 20%), the percent inhibition had no apparent linear relation to the H-TL1 concentration. SPR analysis was used to further confirm the inhibitory effects of H-TL1 on the binding of TNF-α to TNFR1. As expected, TNF-α was strongly bound with the chip-immobilized TNFR1. Further, the elicited response was markedly attenuated in the presence of 0.5 and 1 μM H-TL1 (Figure 1C). These results demonstrate that H-TL1 competitively inhibits the interaction of TNF-α with TNFR1 and has a higher inhibitory ability against TNF-α/TNFR1 than against TNF-α/TNFR2.

### 2.3. H-TL1 Reduced the Effects of TNF-α In Vitro

**Figure 2:**
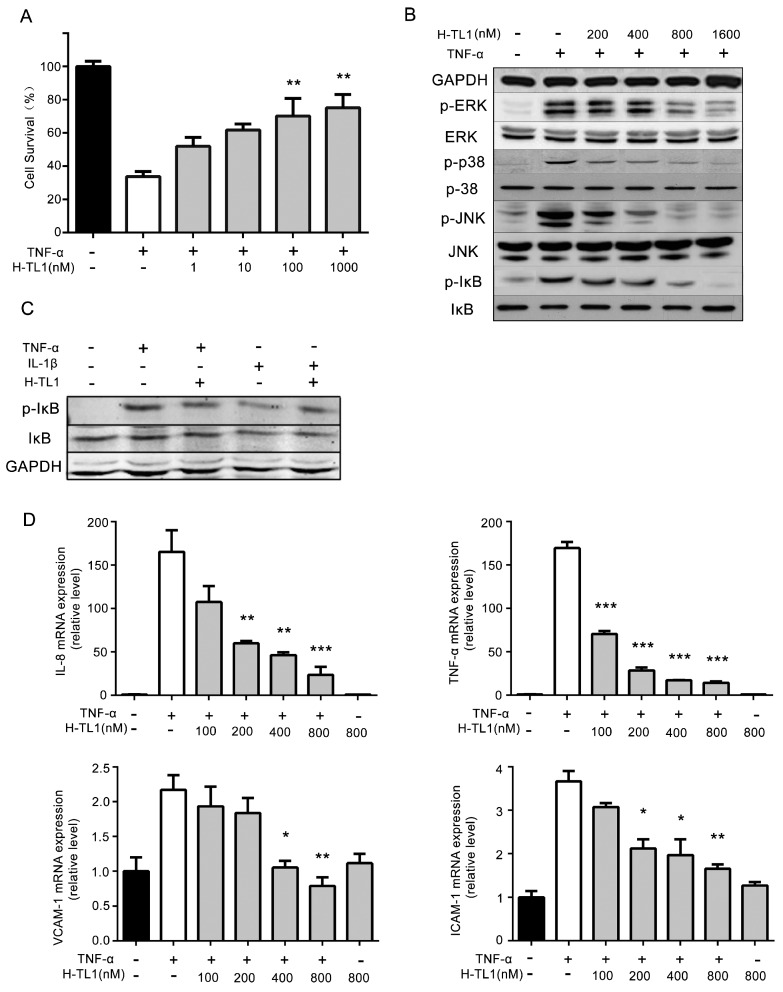
H-TL1 reduced the effects of TNF-α in vitro: (**A**) H-TL1 improved survival in TNF-α-treated L929 cells; (**B**) H-TL1 inhibited the TNF-α-induced phosphorylation of p38 (Thr180/Tyr182), ERK (Thr202/Tyr204), JNK (Thr183/Tyr185), and IκB (Ser32/36) within the NF-κB and MAPKs signaling pathways; (**C**) H-TL1 failed to inhibit the phosphorylation of IκB induced by IL-1β; and (**D**) H-TL1 inhibited TNF-α-mediated expression of pro-inflammatory factors. HEK293 cells were stimulated with TNF-α (20 ng/mL) for 10 min with graded concentrations of H-TL1. Values represent means ± SEM (A: *n* = 5; D: *n* = 3), * *p* < 0.05, ** *p* < 0.01 and *** *p* < 0.001 vs. TNF-α group.

We used the L929 cell line, which is commonly chosen by researchers, to evaluate the effects of H-TL1 on TNF-α-mediated cytotoxicity [23,24]. The cells were treated with TNF-α and 0.2 μg/mL actinomycin D in the presence of graded concentrations of H-TL1. Cell viability was evaluated using MTT assay. As shown in Figure 2A, H-TL1 inhibited TNF-α-induced L929 cell death in a concentration-dependent manner from 1 to 1000 nM. Furthermore, H-TL1 was not cytotoxic against L929 cells at 100 μM (data not shown), which far exceeds the concentration of H-TL1 used in the in vitro assays.

The NF-κB and MAPKs pathways are key signaling pathways of TNF-α-mediated inflammation [1]. Therefore, we analyzed the mechanisms underlying H-TL1-induced inhibition of TNF-α-mediated inflammation by Western blotting in HEK-293 cells. The Western blotting results show that TNF-α-mediated IκB (Ser32/36) and JNK (Thr183/Tyr185) phosphorylation is suppressed by 0.4–1.6 μM H-TL1, whereas phosphorylation of p38 (Thr180/Tyr182) and ERK (Thr202/Tyr204) is inhibited by 0.8–1.6 μM (Figure 2B). To identify the specific effects of H-TL1 on TNF-α downstream signaling, we induced IL-1β as a potent activator of NF-κB that is specific to that pathway. As shown in Figure 2C, H-TL1 failed to inhibit IL-1β-induced phosphorylation of IκB.

NF-κB activation through TNF-α/TNFR interactions upregulates the expression of chemokines and adhesion factors such as IL-8, TNF-α, ICAM-1, and VCAM-1 [25,26]. To determine whether H-TL1 inhibits TNF-α-mediated pro-inflammatory activity, we further assessed the inhibitory activity of H-TL1 on TNF-α-induced expression of these genes in HEK-293 cells. We found that H-TL1 suppresses TNF-α-induced expression of IL-8, TNF-α, ICAM-1, and VCAM-1 in a concentration-dependent manner (Figure 2D). These results provide further evidence of the in vitro anti-inflammatory activity of H-TL1.

### 2.4. H-TL1 Alleviated Experimental Colitis in Mice

IBD represents a group of idiopathic, chronic, and inflammatory intestinal conditions. Anti-TNF-α antibodies are effective in treating IBD that is unresponsive to standard treatments [7,27]. DSS-induced colitis correlates well with human IBD and is considered a suitable model for investigating the pathogenesis and potential therapeutic options for IBD [28]. H-TL1 effectively inhibited TNF-α-mediated pro-inflammatory signaling in vitro, implying that it might be of therapeutic use in inflammatory diseases. Therefore, we examined the anti-inflammatory activity of H-TL1 in vivo using a DSS-induced colitis murine model. Symptoms associated with DSS-induced colitis significantly improved in mice treated with H-TL1 (1 mg/kg) compared to those of vehicle-treated mice.

**Figure 3:**
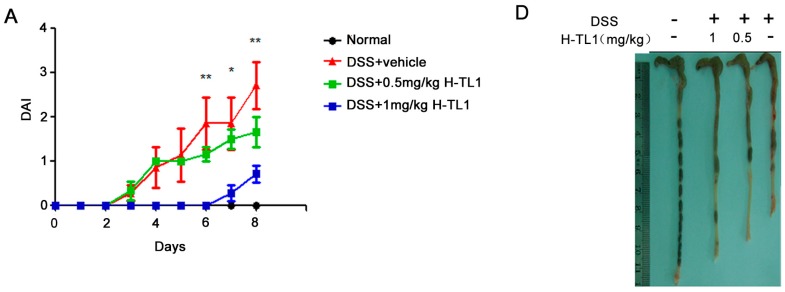

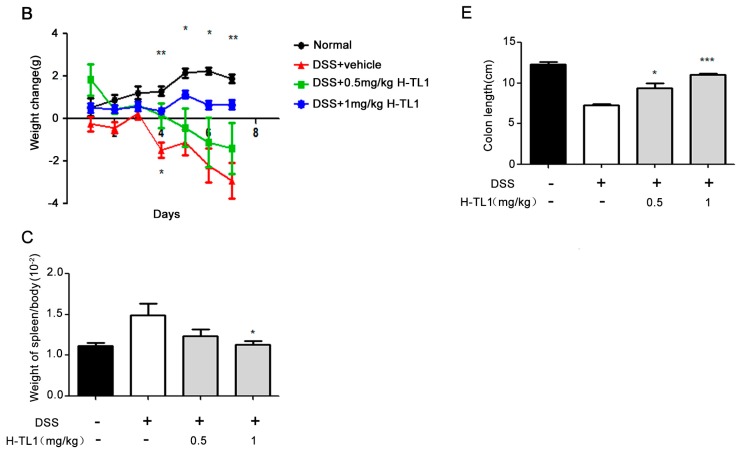
H-TL1 ameliorated DSS-induced colitis: (**A**) Effect of H-TL1 on DAI induced by DSS. DAI (fecal bleeding, diarrhea, and weight loss) was monitored for eight days; (**B**) Effect of H-TL1 on body weight of mice with DSS-induced acute colitis. Values represent means ± SEM (*n* = 8), * *p* < 0.05 and ** *p* < 0.01 vs. DSS group (upper panels) for group treated with H-TL1 (1 mg/kg); * *p* < 0.05 vs. DSS group (lower panels) for group treated with H-TL1 (0.5 mg/kg); (**C**) Effects of H-TL1 on spleen/body weight ratio in mice with DSS-induced acute colitis; (**D**,**E**) Effects of H-TL1 on colon length in mice with DSS-induced acute colitis. The DSS-negative group was considered normal. In (**A**,**C**,**E**), values represent means ± SEM (*n* = 8), * *p* < 0.05, ** *p* < 0.01, and *** *p* < 0.001 vs. DSS group.

The clinical disease activity index (DAI, Table 1) was monitored daily for the entire experiment. As shown in Figure 3A, DSS administration significantly induced diarrhea, rectal bleeding, and weight loss. However, by Days 6–8, the DAI significantly decreased in mice administered 1 mg/kg H-TL1 intraperitoneally once daily (* *p* < 0.05, ** *p* < 0.01). By Day 8, the DSS + vehicle-treated mice lost an average of 4.3 g more than the normal group (without DSS). However, mice treated with H-TL1 were protected from this effect, experiencing only a 1.6 g loss of body weight. Further, DSS + H-TL1 (1 mg/kg) mice recovered, appearing similar in health to that of the normal group (Figure 3B). In addition, the spleen/body weight ratio was significantly lower in the group treated with 1 mg/kg of H-TL1 than that of mice treated with DSS alone (* *p* < 0.05, Figure 3C); however, the 0.5 mg/kg dose did not significantly prevent losses in body weight.

DSS-induced colon inflammation shortened the colon length of the mice (Figure 3D). Treatment with 0.5 and 1 mg/kg H-TL1 significantly attenuated the reduction in colon length (Figure 3D,E, * *p* < 0.05 and *** *p* < 0.001 respectively).

### 2.5. Effects of H-TL1 on Histopathology, Cytokines, and Signaling Pathways in Colonic Mucosa

**Figure 4:**
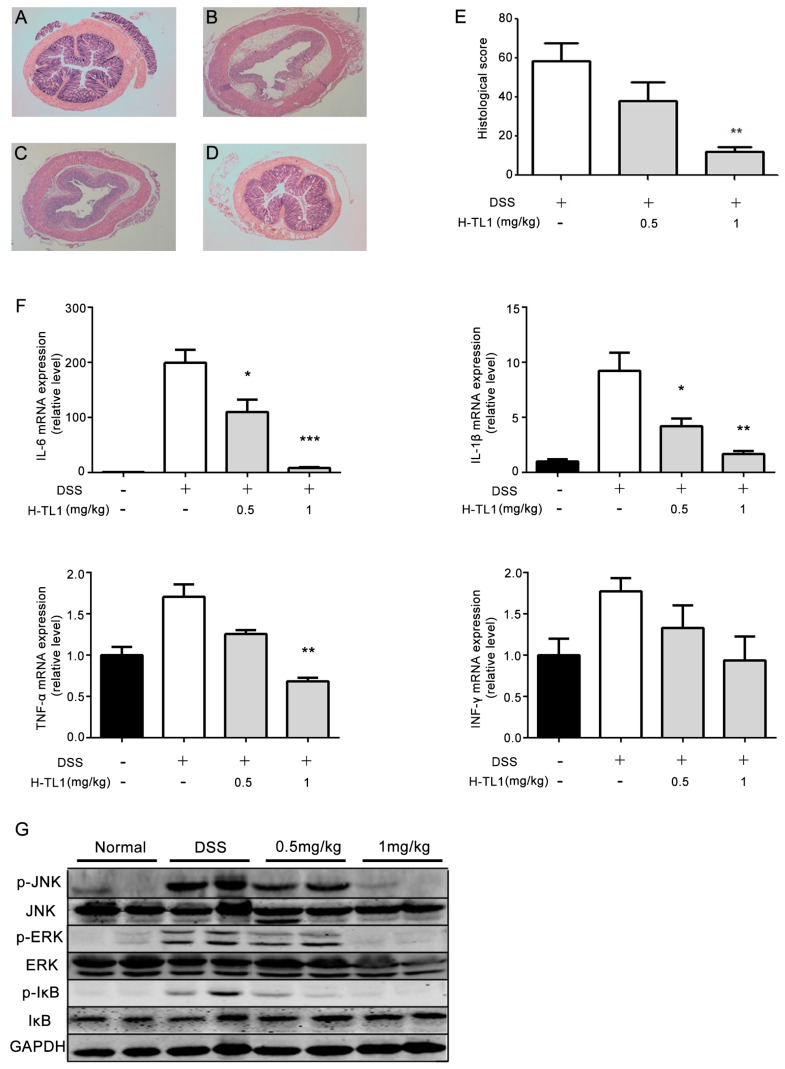
The effect of H-TL1 on inflammatory parameters: histological score, cytokines, and signaling pathways: (**A**) normal mucosa of BALB/c mouse; (**B**) severe inflammation, including complete destruction of epithelial architecture with loss of crypts and epithelial integrity, submucosal edema, and extensive inflammatory cellular infiltration in DSS + vehicle mice; (**C**) treatment with 0.5 mg/kg H-TL1 had little effect on DSS-induced colonic inflammation; (**D**) treatment with 1 mg/kg H-TL1 alleviated DSS-induced colonic inflammation; (**E**) histological score of DSS-induced colonic inflammation in mice treated with vehicle or H-TL1; (**F**) H-TL1 treatment reduced mRNA expression of inflammatory mediators; and (**G**) H-TL1 inhibited DSS-induced NF-κB and MAPK activation through phosphorylation of ERK(Thr202/Tyr204), JNK (Thr183/Tyr185), and IκB (Ser32/36). Values represent means ± SEM (**E**: *n* = 8; **F**: *n* = 3), * *p* < 0.05, ** *p* < 0.01, and *** *p* < 0.001 vs. DSS only group. H&E (hematoxylin and eosin) staining, 40× magnification.

Evaluation of colon morphology indicated that DSS induced extensive damage, including complete destruction of epithelial architecture with loss of crypts and epithelial integrity, submucosal edema, and extensive inflammatory cellular infiltration (Figure 4B). These effects were remarkably alleviated by administration of 1 mg/kg H-TL1, especially the reconstruction of crypts and epithelium (Figure 4D). However, the group administered 0.5 mg/kg H-TL1 had serious colonic damage that was comparable to that of the DSS + vehicle-treated mice (Figure 4C). The total histological score showed that 1 mg/kg H-TL1 significantly reduces DSS-induced inflammation of colonic mucosa (** *p* < 0.01, Figure 4E). 

Chronic mucosal inflammation in IBD is characterized by hyper-activation of immune cells that produce high levels of pro-inflammatory cytokines, resulting in colonic tissue damage [29,30]. Therefore, to gain further insight into the molecular mechanisms underlying the suppression of colitis by H-TL1, we investigated its effects on the mRNA expression of pro-inflammatory cytokines. DSS administration induced aberrantly high expression of TNF-α, IFN-γ, IL-1β, and IL-6 (from 1.73- to 41-fold increases), while treatment with 1 mg/kg, but not 0.5 mg/kg, H-TL1 significantly reduced these effects (Figure 4F). 

NF-κB and MAPKs are key regulatory pathways involved in the development of colitis. To elucidate the underlying intracellular signaling events associated with the effects of H-TL1, two separate colonic tissue extracts from DSS mouse models treated with the vehicle or H-TL1 were subjected to Western blotting. In the group treated with DSS, JNK, ERK, and IκB were activated; however, this effect was inhibited by H-TL1 treatment. Phospho-IκB (Ser32/36), the downstream target of TNF-α/TNFR1, was inhibited by 0.5 mg/kg H-TL1 (Figure 4G). Based on these findings, we suggest that H-TL1 exerts its therapeutic effects by significantly inhibiting TNFR1-mediated cytotoxicity and inflammatory responses in colonic epithelial mucosa, thereby alleviating DSS-induced wasting disease.

### 2.6. H-TL1 Reduced Mortality of Mice with LPS-Induced Acute Shock and Pathological Alterations

**Figure 5:**
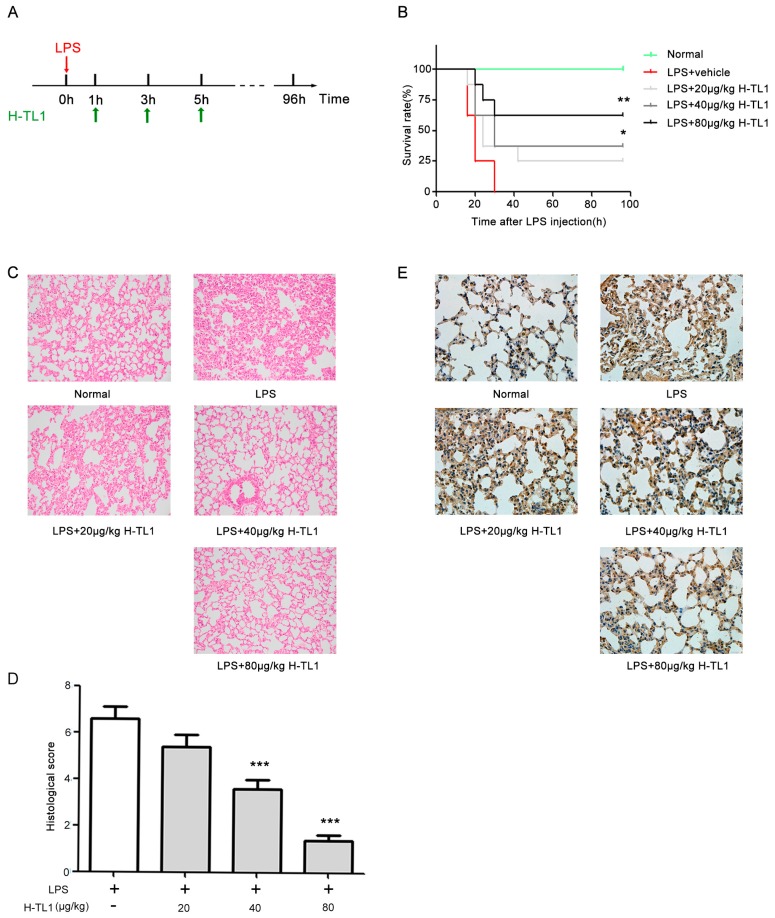
Protective effects of H-TL1 against LPS-induced acute shock in mice: (**A**) timeline of acute septic shock mouse model and treatment with H-TL1; (**B**) survival rates of mice were measured within 96 h. Group without LPS was considered normal, * *p* < 0.05 and ** *p* < 0.01 vs. LPS group (log-rank test); (**C**) effects of H-TL1 on LPS-induced lung tissue histological damage; (**D**) histological score of lung tissue samples; and (**E**) effects of H-TL1 on LPS-induced expression of TNF-α in lung tissue samples. Values represent means ± SEM (*n* = 8), *** *p* < 0.001 vs. LPS group (ANOVA). H&E staining, 200× magnification; IHC, 400× magnification.

Sepsis is considered an excessive systemic pro-inflammatory reaction to invasive microbial pathogens, and acute shock is the most severe symptom associated with sepsis. Cytokines are extensively released during systemic sepsis, including TNF-α, which has been widely reported to be an important mediator of severe sepsis and acute septic shock [31,32,33]. Inhibition of TNF-α significantly prolongs the life span of mice with LPS-induced acute shock. In this study, the mice were observed every 4 h after treatment with LPS (Figure 5A). As shown in Figure 5B, mice treated with LPS + vehicle all died within 30 h, whereas administration of H-TL1 at 40 and 80 μg/kg significantly increased the survival rate to 37.5% and 62.5% (* *p* < 0.05 and *** *p* < 0.001,) respectively. Moreover, H-TL1 protected the mice from LPS-induced acute shock in a dose-dependent manner.

Acute lung injury is a major adverse consequence of acute shock. To assess whether H-TL1 protects mice from LPS-induced lung tissue damage, histological analyses were performed to examine pathological alterations in the lung. Severe damage was observed in lung tissue 12 h after LPS injection, as evidenced by marked tissue edema, congestion, infiltration of inflammatory cells, and hemorrhage (Figure 5C). However, administration of H-TL1 markedly reduced lung parenchymal damage and inflammatory cell infiltration (Figure 5C,D). To further evaluate the effects of H-TL1 on acute shock, we examined the expression of TNF-α in lung tissue using immunohistochemistry (IHC). In the LPS group, TNF-α expression markedly increased; however, administration of H-TL1 attenuated this effect (Figure 5E).

## 3. Materials and Methods

### 3.1. Reagents

The T7Select1-2b display vector, T7 select primers, blocking reagent, and elution buffer were purchased from Novagen (Merck, Kenilworth, NJ, USA). Human recombinant TNF-α (hrTNF-α, 17.4 kDa), soluble TNFR1 (sTNFR1, 18.3 kDa), soluble TNFR2 (sTNFR2, 18.9 kDa), and IL-1β were purchased from PeproTech (Rocky Hill, NJ, USA). DSS (36–50 kDa) was bought from MP Biomedicals (Solon, OH, USA). MTT, actinomycin D, and LPS were purchased from Sigma-Aldrich (St. Louis, MO, USA).

### 3.2. Biological Materials

The *H. cyanocinctus* specimens were captured during September 2005 in the South China Sea near Guangdong Province, People’s Republic of China. After collection, the snakes were killed, and the venom glands were isolated and immediately frozen in liquid nitrogen until use. HEK-293 human embryonic kidney and L929 mouse fibroblast cell lines were obtained from ATCC and cultured in Dulbecco’s modified Eagle’s medium (DMEM, Sigma-Aldrich, St. Louis, MO, USA) containing 10% (*v*/*v*) heat-inactivated fetal bovine serum (FBS, Gibco, BRL, Grand Island, NY, USA) at 37 °C in an atmosphere of 5% CO_2_. The BLT5403 *E. coli* strain (used as the phage host strain) was from Novagen (Merck) and was cultured at 37 °C for propagation. The specific pathogen-free (SPF) male BALB/c and C57BL/6 mice (both 6–8 weeks old), weighing 20 ± 2 g were purchased from Shanghai SLAC Laboratory Animal Co., Ltd. (Shanghai, China). The mice were housed in animal facilities with 50% humidity under a 12-h light-dark cycle, fed a standard pellet diet, and provided with tap water ad libitum. The animal experimental protocols in this research, including venom gland isolation and the mouse study, were approved by the Animal Care and Use Committee of the Second Military Medical University.

### 3.3. RNA Isolation and Construction of T7 Phage Display Library

Total RNA was extracted from fresh *H. cyanocinctus* venom glands using Trizol reagent (Takara Bio., Kusatsu, Japan) and purified using Straight A’s mRNA Isolation System (Novagen, Kenilworth, NJ, USA). The cDNA library was constructed with the T7Select 10-3 OrientExpress cDNA Cloning System, oligo (dT) (Novagen). The recombinant vectors were subsequently packaged with 25 μL T7 Packaging Extract, and the phages were propagated in *E. coli* BLT5403. Finally, the packaged phage library titer was determined with a plaque assay, and the amplified library was stored at −80 °C in 10% glycerol.

### 3.4. Screening and Identification of TNF-α-Binding Peptides Using Phage Display

The phage biopanning procedures for identifying TNF-α-binding peptides were performed on the *H. cyanocinctus* venom gland T7 phage display library. Briefly, 1.0 × 10^9^ phages were added to a 96-well plate (Nunc, Thermo Fisher Scientific, Waltham, MA, USA) coated with hrTNF-α at 1 μg/mL, and the nonspecific sites were blocked with 5% blocking reagent (Novagen). The plates were then incubated for 1 h at 37 °C. Unbound phages were removed by washing the plate four times with Tris-buffered saline (TBS) containing 0.5% Tween-20 (TBST). Then, the bound phages were eluted with 200 μL elution buffer (Novagen) and propagated in the exponentially growing *E. coli* BLT5403 strain for 12 h at 37 °C. The amplified phages were then centrifuged for 15 min at 8000× *g*. The supernatant was collected and used in the next cycle. Elution buffer titers and amplified phages were quantified after each selection by plating serial dilutions of phages on LB agar plates. Briefly, 250 μL of log-phase *E. coli* BLT5403 cells were infected with 100 μL of phage dilutions, mixed with LB top agarose (LB with 0.6% agarose), and then immediately poured onto a pre-warmed LB agar plate. After 3–4 h, the phage plaques formed on the plate were counted and subsequently converted to phage titers. After three rounds of selection, plates with plaques from the phage titration were used to randomly pick individual clones. The plaques were scraped from the plates using a sterile pipette tip and stored at 4 °C in a sterile tube in 10 μL extraction buffer. The individual phages in the plaques were amplified in *E. coli* BLT5403 cells at 37 °C for 1–3 h or until the cells lysed. After cell lysis, the bacteria were removed by centrifugation at 8000× *g* for 10 min. The displayed proteins or peptides of the individual phages were identified by sequencing the corresponding gene fragment presented in the phage DNA. Therefore, PCR was performed using individual phage clones as DNA template and T7 select primers (forward, 5′-AACCCCTCAAGACCCGTTTA-3′ and reverse, 5′-GGAGCTGTCGTATTCCAGTC-3′). The fusion genes of the selected phages were sequenced, and the nucleotide sequences with corresponding amino acid sequence of the peptide were subsequently obtained.

### 3.5. Peptide Synthesis

H-TL1 was synthesized by the peptide synthesizer APeptide Co., Ltd. (Shanghai, China) and purified up to 95%. The lyophilized powder was freshly dissolved in normal saline before use.

### 3.6. ELISA Evaluation

ELISA plates were coated with sTNFR1 (100 ng/well in 100 μL of 100 mM sodium carbonate, pH 9.6) for 20 h at 4 °C, followed by blocking with phosphate buffered saline (PBS) containing 0.2% Tween-20 (PBST) and 1% BSA for 1 h at 37 °C. Different concentrations of H-TL1 (0–1 μM) were mixed with hrTNF-α (20 ng/mL) in PBS containing 1% BSA, and 100 μL/well was placed in the ELISA plates and incubated for 1 h at 37 °C. After washing with PBST, an HRP-labeled monoclonal antibody against TNF-α was added, followed by incubation at 37 °C for 1 h. The plates were then washed, incubated with *o*-phenylenediamine, and the absorbance was subsequently measured at 450 nm.

### 3.7. SPR Analysis

sTNFR1 (100 μg/mL) was covalently immobilized onto a CM5 sensor chip by the BIAcore T200 system (Biacore, GE Healthcare) following manufacturer’s instructions. To determine whether H-TL1 inhibits the binding of TNF-α to TNFR1, hrTNF-α was diluted in running buffer at 100 nM with or without different concentrations of H-TL1 (0.1, 0.5, and 1 μM) and subsequently passed over the sTNFR1-immobilized chip for 360 s, followed by dissociation for 200 s. The response unit indicates binding ability.

### 3.8. MTT Assay

For MTT cell viability assay, L929 cells were seeded onto a 96-well plate and cultured for 12 h. Then, serial dilutions of H-TL1 were prepared in medium containing 20 ng/mL hrTNF-α and 0.2 μg/mL actinomycin D and applied to cells. Following incubation at 37 °C for 18–20 h, MTT solution was added, followed by incubation at 37 °C for 4 h, and then the absorbance was determined at 490 nm. Blank control wells containing cell culture medium only and normal control wells with untreated cells were used to normalize the cell viability calculation.

### 3.9. Western Blot Analysis

For Western blotting, the cell or tissue specimen extracts were resolved in a 10% SDS-PAGE gel, and then the separated proteins were transferred to a nitrocellulose filter membrane. The antigens were detected by using specific antibodies against total and phosphorylated IκB, p38, ERK, and JNK followed by incubation with the respective secondary antibodies. GAPDH was simultaneously detected as the control to compare the protein loading in each lane. The mouse monoclonal anti-IκB, mouse monoclonal anti-p-IκB (Ser32/36), rabbit monoclonal anti-ERK, and rabbit monoclonal anti-p-ERK (Thr202/Tyr204) antibodies were from Cell Signaling Technologies (Beverly, MA, USA). The mouse polyclonal anti-p-p38 (Thr180/Tyr182), rabbit polyclonal anti-p38, rabbit monoclonal anti-p-JNK (Thr183/Tyr185), and mouse monoclonal anti-JNK antibodies were from Thermo Fisher Scientific (Rockford, IL, USA). The rabbit monoclonal anti-GAPDH antibody was from Santa Cruz Biotechnology (Santa Cruz, CA, USA), and the fluorescent-labeled secondary antibodies were from Li-COR Biosciences (Lincoln, NE, USA).

### 3.10. RNA Extraction and Real-Time PCR

Total RNA was extracted from colon tissue specimens or cells using Trizol reagent (Takara) according to the manufacturer’s instructions and was then reverse transcribed using random primers. Real-time PCR was performed on a StepOne Real-Time PCR System (Applied Biosystems, Foster City, CA, USA) to analyze the mRNA expression levels of IL-8, TNF-α, IL-1β, IL-6, VCAM-1, and ICAM-1. The relative fold changes of target gene expression were comparatively quantified by the 2^−ΔΔ*C*t^ method [34]. GAPDH was chosen as the internal control gene, and samples without treatment were designated as the comparing reference. Primers used in this study were based on published sequences [6,35,36].

### 3.11. Induction of Colitis and Treatments

To induce colitis, 6–8 week old male BALB/c mice were administered 2.5% DSS solution in their drinking water for 7–8 consecutive days, ad libitum. Importantly, 30–40 kDa DSS was used to establish the animal model in this study because it is known to induce more severe colitis than 5 and 500 kDa DSS [37]. The mice were concomitantly administered H-TL1 (0.5 or 1 mg/kg) once daily for 7–8 consecutive days by intraperitoneal injection. Establishment of the DSS-induced colitis was evidenced by an increase in DAI, with associated weight loss, loose stool consistency, and presence of blood in the feces [38]. DAI was monitored during the entire experiment and was assessed according to the method described in Table 1.

### 3.12. LPS-Induced Acute Shock and Treatments

The LPS-induced acute shock model was established in male C57BL/6 mice (6–8 weeks old) that were randomly allocated to five groups. LPS was administered (15 mg/kg body weight) to induce acute shock. The LPS- or normal saline-treated mice were injected with 100 μL of normal saline with or without LPS intraperitoneally, while the H-TL1 and LPS-treated mice were each administered H-TL1 at three doses of 20, 40, and 80 μg/kg 1, 3, and 5 h after the LPS injection (Figure 5A). The mice were observed every 4 h to evaluate mortality.

### 3.13. Colon Histopathology

The colonic tissue samples were fixed in 4% neutral formalin, dehydrated with increasing concentrations of ethanol, embedded in paraffin, and subsequently cut into 5-μm thick sections, which were mounted on slides, cleared, hydrated, and stained with hematoxylin and eosin (H&E). Histological grading was assessed according to previously described criteria (Table 2) [39], and the scoring criteria used were similar to those described previously [40]. 

### 3.14. Lung Histopathology

To assess lung damage, lung tissues were excised, inflated with 4% neutral formalin, and H&E stained 12 h following LPS injection. The lung parenchyma was examined by light microscopy and graded based on the degree of congestion, edema, inflammation, and hemorrhage. Each characteristic was scored on a scale of 0 to 3 (0, absent; 1, light; 2, moderate; and 3, strong).

### 3.15. Immunohistochemistry

An additional set of paraffin sections was used for immunohistochemical analysis of TNF-α expression. Sections (5-μm thick) were mounted on slides, cleaned, hydrated, blocked with endogenous peroxidase (Dako, S2001), washed with PBS, and then treated with citrate buffer (10 nM, pH 6.0) for target antigen retrieval. The sections were treated with a buffered blocking solution (3% BSA in PBS for 15 min), incubated for 1 h at room temperature with a primary polyclonal antibody specific to mouse TNF-α, washed with PBS, and incubated with a secondary HRP-labeled antibody (Abcam) (1:500 in PBS, *v*/*v*) at room temperature for 1 h. Then, the sections were washed again with Tris-HCl 0.05 M, pH 7.6, and subsequently incubated with 3,3′-diaminobenzidine (DAB) solution in the dark at room temperature for 10 min. The sections were finally washed with Tris-HCl and counterstained with hematoxylin.

### 3.16. Statistical Analysis

The multiple comparison data are presented as the means ± SEM and were assessed by one-way analysis of variance (ANOVA). In survival curve comparisons, data were transformed using a Mantel-Cox log-rank test. All the statistical analyses were conducted using GraphPad Prism 5 software for Windows (GraphPad Software, San Diego, CA, USA), and *p* < 0.05 was considered statistically significant.

## 4. Conclusions

Snake venom is primarily composed of proteins and peptides that possess a variety of biological activities. In addition, venom has been shown to have pro- and anti-inflammatory properties and has been shown to regulate the expression of inflammatory mediators such as IL-1β, IL-6, IL-10, IL-22, TNF-α, PGD2, PGE2, and TXA2 [18,19,21,41,42,43,44]. In this study, we constructed and screened an *H. cyanocinctus* venom gland T7 phage display library and discovered H-TL1, a natural peptide with antagonizing effects against TNF-α. We subsequently evaluated the anti-inflammatory effects of H-TL1. The in vitro results demonstrate that H-TL1 specifically disrupts formation of the TNF-α/TNFR1 complex, blocks the phosphorylation of well-characterized TNF-α/TNFR1 downstream signaling transduction pathways, and suppresses TNFR1-mediated mRNA expression of pro-inflammatory cytokines, including TNF-α. Additionally, H-TL1 treatment resulted in little cytotoxicity toward mammalian cells. In vivo data from two models of inflammation, DSS-induced colitis and LPS-induced acute shock, revealed that treatment with H-TL1 at the onset of disease markedly ameliorates the clinical manifestations and inflammation-related pathologic damage to the mouse colonic mucosa and lung parenchyma. Furthermore, in accordance with the cell experiments, treatment with H-TL1 inhibited TNF-α-mediated activation of NF-κB and MAPKs pathways and reduced the production of inflammatory mediators in injured tissues.

In summary, H-TL1, screened from an *H. cyanocinctus* venom gland T7 phage display library, effectively antagonized the TNF-α/TNFR1 interaction and alleviated the cytotoxicity and inflammation associated with TNF-α in vitro and in vivo. These new findings emphasize the potential usefulness of H-TL1 as a promising peptide candidate for the development of new agents to treat IBD, sepsis acute shock, and other inflammatory diseases associated with TNF-α.

## Figures and Tables

**Table 1:** Criteria for scoring disease activity index (DAI).

Scores	Body Weight Loss (%)	Stool Consistency	Presence or Absence of Fecal Blood
0	none	well-formed pellets	none
1	1–5	well-formed pellets	none
2	5–10	loose stools	slight bleeding
3	10–20	loose stools	slight bleeding
4	>20	diarrhea	gross bleeding

**Table 2:** Histological score.

Scores	Severity of Inflammation	Extent of Inflammation	Crypt Damage
0	none	none	none
1	mild	mucosal	basal 1/3
2	moderate	mucosal and submucosal	basal 2/3
3	severe	transmural	crypts lost but surface epithelium present
4	-	-	crypts and surface epithelium lost

## Abbreviations

TNF-α	Tumor necrosis factor-α
IBD	Inflammatory bowel disease
ELISA	Enzyme-linked immunosorbent assay
SPR	Surface plasmon resonance
MTT	3-(4,5-Dimethylthiazol-2-yl)-2,5-diphenyl tetrazolium bromide
DSS	Dextran sodium sulfate
LPS	Lipopolysaccharide
DAI	Disease activity index
IHC	Immunohistochemistry
